# Perioperative Dynamics of the Hemoglobin–Albumin–Lymphocyte–Platelet (HALP) Score and Early Morbidity After Curative Esophagectomy

**DOI:** 10.3390/jcm15218716

**Published:** 2026-11-08

**Authors:** Hürü Ceren Gökduman, Oğuzhan Aytepe, Yiğit Düzköylü, Sercan Yüksel, Hüsnü Ozan Şevik, Taner Abdullah, Mert Şentürk, Funda Gümüş Özcan

**Affiliations:** 1 Department of Anesthesiology, Basaksehir Cam and Sakura City Hospital, Istanbul 34480, Türkiye; cerengokduman@hotmail.com (H.C.G.); taner.abdullah@gmail.com (T.A.); fgumus@hotmail.com (F.G.Ö.); 2 Department of General Surgery, Basaksehir Cam and Sakura City Hospital, Istanbul 34480, Türkiye; aytepeoguzhan34@gmail.com (O.A.); drsercanyuksel@gmail.com (S.Y.); drhusnusevik@gmail.com (H.O.Ş.); 3 Department of Gastroenterological Surgery, Basaksehir Cam and Sakura City Hospital, Istanbul 34480, Türkiye; dryigit@gmail.com; 4 Department of Anesthesiology and Reanimation, School of Medicine, Acibadem Mehmet Ali Aydinlar University, Istanbul 34638, Türkiye

**Keywords:** esophagectomy, HALP score, postoperative complications, immune–nutritional status, perioperative biomarkers, esophageal cancer

## Abstract

**Background/Objectives**: The hemoglobin–albumin–lymphocyte–platelet (HALP) score is an immune–nutritional biomarker associated with oncological outcomes. We evaluated perioperative HALP trajectories and their association with early postoperative complications after curative esophagectomy. **Methods**: In this retrospective single-center cohort study, 129 patients undergoing esophagectomy between June 2020 and March 2025 were included. HALP was calculated preoperatively and on postoperative days 1 and 7. Trajectories were analyzed using generalized estimating equations. Discriminative performance was assessed using receiver operating characteristic (ROC) analysis, and independent associations were evaluated using multivariable logistic regression. **Results**: HALP declined markedly after surgery (median, 28.93 preoperatively, 10.11 on postoperative day 1, and 10.53 on postoperative day 7; *p* < 0.001). Patients with major complications, anastomotic leakage, or intensive care unit readmission had lower postoperative day 7 HALP values. Postoperative day 7 HALP discriminated intensive care unit readmission (area under the curve [AUC] = 0.769), anastomotic leakage (AUC = 0.734), major complications (AUC = 0.733), and overall complications (AUC = 0.697). Each doubling of postoperative day 7 HALP was independently associated with lower odds of overall complications (odds ratio [OR] = 0.473), major complications (OR = 0.351), anastomotic leakage (OR = 0.383), and intensive care unit readmission (OR = 0.374; all *p* ≤ 0.002). Preoperative HALP showed limited discriminative performance. Bootstrap-corrected AUCs for the models including age, ASA, and POD7 HALP ranged from 0.696 to 0.737. **Conclusions**: HALP demonstrates a distinct perioperative trajectory after esophagectomy, with persistent impairment on postoperative day 7 associated with early postoperative morbidity. The potential clinical role of serial HALP assessment requires prospective evaluation.

## 1. Introduction

Esophageal cancer remains one of the most aggressive gastrointestinal malignancies worldwide and continues to be associated with poor long-term survival despite advances in multimodal treatment. For patients with localized disease, esophagectomy following neoadjuvant therapy remains the cornerstone of curative treatment. However, esophagectomy is one of the most physiologically demanding oncologic procedures and is associated with postoperative complication rates approaching 50–60%, even in high-volume centers. Pulmonary complications, anastomotic leakage, infectious complications, and prolonged hospitalization continue to represent major causes of postoperative morbidity and mortality. Consequently, modern perioperative care has evolved beyond technical aspects of surgery toward comprehensive optimization of the patient’s physiological reserve before, during, and after the operation [1,2,3].

Host nutritional status and systemic inflammatory response are now recognized as key determinants of postoperative recovery after esophagectomy. Malnutrition impairs wound healing, immune competence, and preservation of lean body mass, whereas persistent systemic inflammation promotes catabolism, endothelial dysfunction, and postoperative organ injury. Reflecting this evidence, current Enhanced Recovery After Surgery (ERAS) and European Society for Clinical Nutrition and Metabolism (ESPEN) guidelines emphasize routine nutritional screening, correction of nutritional deficiencies, prehabilitation, attenuation of surgical stress, and early postoperative nutritional support as integral components of perioperative management for patients undergoing esophageal cancer surgery [2,3,4,5]. Likewise, optimization of perioperative anemia has become an important strategy to improve oxygen delivery and reduce postoperative complications [6].

Given the close interaction between nutritional status, immune competence, and systemic inflammation, several composite biomarkers have been proposed for perioperative risk stratification. Among these, the hemoglobin–albumin–lymphocyte–platelet (HALP) score combines four routinely available laboratory parameters reflecting oxygen-carrying capacity, nutritional reserve, immune function, and inflammatory activity into a single immune–nutritional index. Since its introduction, the HALP score has demonstrated prognostic value in several gastrointestinal malignancies, particularly esophageal and gastric cancers, while recent systematic reviews and meta-analyses have further supported its broader applicability across digestive system cancers. Lower pretreatment HALP scores have consistently been associated with inferior overall survival, disease-free survival, and recurrence-free survival, suggesting that HALP reflects host biological status rather than tumor burden alone [7,8,9,10,11,12].

Despite these promising findings, current evidence has almost exclusively evaluated HALP as a static preoperative biomarker for long-term oncologic outcomes. Whether this approach adequately reflects perioperative physiological changes remains uncertain. Esophagectomy induces a profound systemic response characterized by acute inflammation, increased vascular permeability, perioperative blood loss, hemodilution, redistribution of albumin, lymphocyte suppression, and marked metabolic stress. Accordingly, each component of the HALP score changes substantially during the postoperative period, potentially providing a more comprehensive representation of the host response to surgical injury than preoperative measurements alone [1,3,13,14].

Increasing evidence indicates that postoperative inflammatory and nutritional trajectories may be more informative than isolated baseline measurements for predicting short-term clinical outcomes after major gastrointestinal surgery. Dynamic postoperative changes in inflammatory biomarkers, serum albumin, and metabolic recovery have all been associated with postoperative complications following esophagectomy [3,13,14]. Furthermore, contemporary perioperative pathways—including prehabilitation, nutritional optimization, perioperative anemia management, and ERAS-based care—modify patients’ physiological status before surgery [2,4,5,6]. Consequently, postoperative immune–nutritional assessment may better integrate baseline physiological reserve with the biological consequences of surgical stress, thereby providing a more comprehensive assessment of the patient’s early recovery than preoperative evaluation alone.

Although the prognostic significance of preoperative HALP has been demonstrated in patients with esophageal cancer, evidence regarding the clinical relevance of perioperative HALP dynamics remains scarce. In particular, whether postoperative HALP provides superior discrimination of patients at risk for early postoperative complications after curative esophagectomy has not been adequately investigated [9,12].

Therefore, the present study aimed to evaluate perioperative HALP trajectories using serial preoperative and postoperative measurements in patients undergoing curative esophagectomy and to determine their association with early postoperative complications. We hypothesized that postoperative HALP would better reflect the evolving physiological response to surgical stress and therefore provide greater predictive value for postoperative morbidity than preoperative HALP alone.

## 2. Materials and Methods

### 2.1. Study Design and Patient Selection

This retrospective cohort study was conducted at Başakşehir Çam and Sakura City Hospital after approval by the Institutional Ethics Committee (Approval No. 2026-206). The June 2020–March 2025 interval refers to the dates of clinical operations; retrospective review of patient records for this research commenced after ethics approval on 5 June 2026. ClinicalTrials.gov Identifier: NCT07761975; retrospectively registered on 23 July 2026. Informed consent was waived because of the retrospective design. The study was conducted in accordance with the Declaration of Helsinki.

Adults (≥18 years) who underwent elective curative-intent esophagectomy for histologically confirmed esophageal squamous cell carcinoma between June 2020 and March 2025 were identified from the institutional electronic medical record system. All patients received neoadjuvant treatment.

A total of 139 consecutive patients were assessed for eligibility. Patients were excluded if they underwent reoperation within the first 7 postoperative days, died before POD7, had missing laboratory data precluding HALP calculation, had hematological disorders potentially affecting HALP interpretation (e.g., essential thrombocythemia), or underwent non-standard procedures substantially altering the extent of resection, such as combined esophagectomy and total laryngectomy. Patients who died or required reoperation before POD7 were excluded because the primary analysis was designed to characterize perioperative HALP trajectories using serial measurements through POD7. Patients without the laboratory data required to calculate HALP at the predefined time points were also excluded from the longitudinal analysis. Ultimately, 129 patients were included in the final analysis (Figure 1). All 129 operations included in the analysis were performed by the same surgical team.

### 2.2. Data Collection

Demographic, clinical, laboratory, operative, and postoperative data were retrieved from electronic medical records. Variables included age, sex, body mass index (BMI), American Society of Anesthesiologists (ASA) physical status, smoking status, comorbidities, pulmonary function tests, preoperative peripheral oxygen saturation, neoadjuvant treatment status, surgical procedure, operative duration, ICU and hospital length of stay, ICU readmission, postoperative complications, anastomotic leakage, and in-hospital mortality. Tumor T, N, and M categories and the corresponding stage-group labels were obtained from the clinical spreadsheet. The treating team identified these entries as post-neoadjuvant pathological staging (ypTNM) under the AJCC eighth-edition framework. Recorded stage-group labels were summarized as supplied, without independent retrospective re-classification.

Postoperative complications were classified according to the Clavien–Dindo classification. Overall complications were defined as grade II or higher, whereas major complications were defined as grade III or higher, including complications requiring surgical, endoscopic, or radiological intervention (grade III), life-threatening complications requiring intensive care management (grade IV), or death (grade V) [15].

### 2.3. HALP Score Assessment

The hemoglobin–albumin–lymphocyte–platelet (HALP) score was calculated according to Chen et al. [16]:

HALP = (Hemoglobin (g/L) × Albumin (g/L) × Lymphocyte count (/L))/(Platelet count (/L))

Hemoglobin concentrations recorded in g/dL were converted to g/L (multiplied by 10) before HALP calculation; lymphocyte and platelet counts were entered in the same units (10^9^/L).

HALP was calculated preoperatively and on POD1 and POD7 using laboratory values closest to each predefined time point. Preoperative, POD1, and POD7 HALP scores were analyzed as continuous variables and according to outcome status.

### 2.4. Outcomes

The primary objective was to evaluate the association between perioperative HALP trajectories and early postoperative outcomes following curative esophagectomy. The primary outcomes included overall postoperative complications (Clavien–Dindo grade II or higher), major complications (Clavien–Dindo grade III or higher), anastomotic leakage, and unplanned intensive care unit (ICU) readmission. Overall and major complications were classified according to the Clavien–Dindo classification. Anastomotic leakage was recorded based on the clinical diagnosis documented in the medical records. Unplanned ICU readmission was defined as an unplanned transfer back to the ICU after initial discharge from the ICU to a lower level of care. Postoperative outcomes were assessed during the index hospital admission. The discriminative performance of HALP at each perioperative time point was also evaluated. Secondary outcomes included ICU length of stay, total hospital length of stay, and in-hospital mortality.

### 2.5. Statistical Analysis

Statistical analyses were performed using IBM SPSS Statistics 27 and R version 4.6.0 (R Foundation for Statistical Computing, Vienna, Austria). Continuous variables were assessed for normality using the Shapiro–Wilk test and graphical inspection. Normally distributed variables are presented as mean ± standard deviation (SD), and non-normally distributed variables as median (interquartile range [IQR]); categorical variables are presented as frequencies and percentages. For recorded T, N, and ypTNM stage groups, percentages were calculated among nonmissing entries (*n* = 128); missing categories were reported separately.

Perioperative changes in HALP and its components were assessed using the Friedman test followed by pairwise Wilcoxon signed-rank tests with Bonferroni correction. Longitudinal associations between HALP trajectories and postoperative outcomes were evaluated using generalized estimating equations (GEE), including time, outcome status, and their interaction as fixed effects and adjusting for age, sex, and ASA physical status, using an exchangeable working correlation structure.

The unadjusted discriminative performance of preoperative, postoperative day 1 (POD1), and postoperative day 7 (POD7) HALP for postoperative outcomes was assessed using receiver operating characteristic (ROC) curve analysis. Areas under the ROC curves (AUCs) were compared using paired DeLong tests, with Holm correction applied for multiple comparisons.

Associations between HALP and binary outcomes were evaluated using multivariable logistic regression. Covariates were selected according to clinical relevance, the outcome being analyzed, and the number of observed events. To limit model complexity for outcomes with relatively few events, parsimonious models were used for anastomotic leakage and unplanned intensive care unit (ICU) readmission, adjusting for age and ASA physical status in addition to the HALP measure of interest. Models for outcomes with more events included additional clinically relevant covariates, as specified in the corresponding tables. HALP effects were expressed per doubling. Discrimination of the fitted multivariable logistic regression models was assessed using apparent AUCs, representing model performance in the derivation sample. Severe complications (Clavien–Dindo grade IV or higher) and in-hospital mortality were not included in multivariable logistic regression models because of the limited number of events (10 and 6, respectively).

Exploratory sensitivity analyses compared the 129 included patients with clinically eligible excluded patients using Mann–Whitney U and Fisher exact tests; preoperative and POD1 HALP associations were also estimated in the expanded cohort. Reduced-covariate logistic models were examined for coefficient stability. For the age-and-ASA clinical models, incremental discrimination after including POD7 HALP was examined by paired DeLong tests and likelihood-ratio tests. Holm adjustments were applied separately within each four-outcome family. Internal validation used 1000 bootstrap resamples to estimate optimism-corrected AUCs, Brier scores, and calibration slopes. For anastomotic leakage and unplanned ICU readmission, Firth penalized logistic regression was additionally fitted using log2 POD7 HALP, age, and numeric ASA class; 95% confidence intervals and *p* values were derived from profile penalized likelihood.

ICU and hospital length of stay were analyzed using negative binomial regression. Correlation between HALP and continuous outcomes were assessed using Spearman’s rank correlation coefficient. All tests were two-sided, with *p* < 0.05 considered statistically significant.

## 3. Results

### 3.1. Clinical and Laboratory Findings

#### 3.1.1. Patient Characteristics

Between June 2020 and March 2025, 139 consecutive patients who underwent curative-intent esophagectomy were assessed for eligibility. After exclusion of 10 patients because of reoperation within POD7 (*n* = 2), death before POD7 (*n* = 1), missing POD7 laboratory data (*n* = 5), essential thrombocythemia (*n* = 1), or combined esophagectomy with total laryngectomy (*n* = 1), 129 patients were included in the final analysis (Figure 1).

Baseline demographic, operative, and postoperative characteristics are summarized in Table 1. All 129 patients (100%) had squamous cell carcinoma and received neoadjuvant treatment. Recorded T, N, and clinician-supplied AJCC (American Joint Committee on Cancer) eighth-edition ypTNM stage-group labels were available for 128 patients; the remaining patient had no recorded T, N, or stage group. M was recorded as M0 for all 129 patients. Recorded stages were I in 64 patients (50.0%), II in 19 (14.8%), III in 32 (25.0%; including IIIA in 21 [16.4%], IIIB in 10 [7.8%], and III without a specified subgroup in 1 [0.8%]), and IVA in 13 (10.2%). The mean age was 58.5 ± 12.1 years, and 63 patients (48.8%) were male. Most patients were classified as ASA II (59.7%) or ASA III (34.9%). McKeown esophagectomy was the most commonly performed procedure (53.5%), with a median ICU stay of 2 (IQR, 1–3) days and a median hospital stay of 19 (IQR, 13–30) days. Overall postoperative complications (Clavien–Dindo grade ≥ II) occurred in 71 patients (55.0%), major complications (grade ≥ III) in 34 (26.4%), anastomotic leakage in 18 (14.0%), ICU readmission in 16 (12.4%), and in-hospital mortality in 6 (4.7%) (Table 1). Intraoperative transfusion categories included erythrocyte suspension (ES), fresh frozen plasma (FFP), or both (Table 1).

#### 3.1.2. Perioperative Changes in HALP

Significant perioperative changes were observed in HALP and all of its individual laboratory components (all *p* < 0.001) (Table 2). Median HALP decreased from 28.93 (IQR, 17.09–44.40) preoperatively to 10.11 (IQR, 6.48–14.76) on POD1 and remained low on POD7 (10.53 [IQR, 6.93–15.02]; Friedman χ^2^(2) = 145.690, *p* < 0.001, Kendall W = 0.565). Albumin and lymphocyte counts demonstrated the largest perioperative changes (Kendall W = 0.666 and 0.579, respectively), whereas platelet count showed the smallest effect size (W = 0.112) (Table 2).

The temporal pattern of HALP is illustrated in Figure 2. HALP declined sharply immediately after surgery and showed only limited recovery by POD7, indicating a sustained postoperative reduction during the first postoperative week.

#### 3.1.3. HALP Trajectories According to Postoperative Outcomes

Generalized estimating equation analysis demonstrated that perioperative HALP trajectories differed according to postoperative outcomes (Table 3). Significant time-by-outcome interactions were observed for major complications (*p* = 0.004), anastomotic leakage (*p* = 0.001), and ICU readmission (*p* = 0.016), whereas the interaction for overall postoperative complications did not reach statistical significance (*p* = 0.148) (Table 3). Holm-adjusted interaction *p* values were 0.012, 0.004, 0.032, and 0.148 for major complications, anastomotic leakage, ICU readmission, and overall complications, respectively.

Across all evaluated outcomes, patients who developed adverse postoperative events consistently exhibited lower HALP values during the postoperative period. At POD7, adjusted geometric mean HALP ratios for patients with versus without complications were 0.646 for overall complications, 0.549 for major complications, 0.555 for anastomotic leakage, and 0.544 for ICU readmission (all *p* < 0.001), demonstrating substantially lower postoperative HALP values among patients experiencing unfavorable clinical outcomes (Table 3).

#### 3.1.4. Association and Discriminative Performance of POD7 HALP for Early Postoperative Outcomes

ROC analysis demonstrated that POD7 HALP consistently discriminated patients who developed adverse postoperative outcomes. Discriminative performance was highest for ICU readmission (AUC = 0.769), followed by anastomotic leakage (AUC = 0.734), major complications (AUC = 0.733), and overall postoperative complications (AUC = 0.697). In contrast, preoperative HALP showed limited discriminatory ability and was not consistently associated with postoperative outcomes (Figure 3).

In paired comparisons, POD7 HALP demonstrated significantly greater discrimination than preoperative HALP for major complications and anastomotic leakage, and greater discrimination than POD1 HALP for anastomotic leakage and ICU readmission after Holm adjustment (Table 4).

Multivariable logistic regression further demonstrated that POD7 HALP remained independently associated with all four prespecified binary postoperative outcomes (Table 5). Each doubling of POD7 HALP was associated with lower odds of Clavien–Dindo grade II or higher complications (adjusted OR, 0.473; 95% CI, 0.299–0.746; *p* = 0.001), major complications (adjusted OR, 0.351; 95% CI, 0.199–0.619; *p* < 0.001), anastomotic leakage (adjusted OR, 0.383; 95% CI, 0.210–0.700; *p* = 0.002), and ICU readmission (adjusted OR, 0.374; 95% CI, 0.201–0.696; *p* = 0.002). The corresponding multivariable models showed apparent AUCs of 0.765, 0.782, 0.723, and 0.774, respectively (Table 4). For anastomotic leakage and unplanned ICU readmission, the respective 18 and 16 events across three slope parameters (log2 POD7 HALP, age, and ASA class entered numerically) corresponded to 6.0 and 5.3 events per parameter. These low ratios were considered when interpreting the sparse-outcome models.

In exploratory analyses of eight clinically eligible excluded patients, POD1 HALP was higher in the seven with available POD1 measurements than in the included group (median 23.75 vs. 10.11; *p* < 0.001). Anastomotic leakage occurred in 3/8 excluded patients and 18/129 included patients (Fisher *p* = 0.105). In the expanded eligible cohort (*n* = 137; POD1 HALP available for *n* = 136), POD1 HALP remained associated with major complications (OR per doubling, 0.604; 95% CI, 0.399–0.914; *p* = 0.017), whereas its association with Clavien–Dindo grade ≥ II was not significant (OR, 0.737; *p* = 0.090). Reduced-covariate POD7 models produced HALP estimates similar to those in Table 5 (overall complications: OR 0.484 vs. 0.473; major complications: OR 0.356 vs. 0.351). Descriptive characteristics of all ten excluded patients are provided in Table 6.

After POD7 HALP was added to age and ASA, the apparent AUCs increased across all four outcomes (Table 7). After Holm adjustment for the four paired DeLong tests, only the ICU readmission AUC difference remained significant (*p* = 0.0129). Bootstrap-corrected AUCs of the HALP-augmented models were 0.698, 0.730, 0.696, and 0.737 for overall complications, major complications, leakage, and ICU readmission, respectively; corresponding calibration slopes were 0.891, 0.909, 0.910, and 0.860. (Figure 4).

AUC values represent apparent same-cohort discrimination (*n* = 129). Holm *p* values refer to the four paired DeLong tests; corrected AUCs are from 1000-resample bootstrap internal validation. These analyses assess recorded complications, not event-free POD7 prediction. Optimism-corrected Brier scores (clinical model versus clinical + POD7 HALP) were 0.247 versus 0.225 for grade ≥ II complications, 0.201 versus 0.175 for major complications, 0.124 versus 0.114 for anastomotic leakage, and 0.113 versus 0.107 for ICU readmission.

For anastomotic leakage, the Firth penalized estimate for POD7 HALP per doubling was OR = 0.406 (95% CI, 0.219–0.707; *p* = 0.0011); for unplanned ICU readmission it was OR = 0.397 (95% CI, 0.210–0.702; *p* = 0.0012). The direction and nominal significance of both estimates were unchanged relative to the conventional logistic models.

In separate models using preoperative HALP instead of POD7 HALP, preoperative HALP was not independently associated with any of the evaluated postoperative outcomes after multivariable adjustment. Each doubling of preoperative HALP was not significantly associated with Clavien–Dindo grade II or higher complications (adjusted OR, 0.721; 95% CI, 0.485–1.073; *p* = 0.107), major complications (adjusted OR, 0.877; 95% CI, 0.572–1.345; *p* = 0.548), anastomotic leakage (adjusted OR, 0.971; 95% CI, 0.575–1.637; *p* = 0.911), or ICU readmission (adjusted OR, 0.645; 95% CI, 0.366–1.136; *p* = 0.129).

Among the additional covariates, intraoperative transfusion was independently associated with an increased risk of overall postoperative complications (adjusted OR, 3.922; *p* = 0.014), whereas female sex was associated with lower odds of major complications (adjusted OR, 0.320; *p* = 0.019). No consistent independent associations were identified for age, ASA physical status, or BMI across the evaluated models.

#### 3.1.5. Secondary Clinical Outcomes

In adjusted count models, higher preoperative HALP was independently associated with shorter ICU stay. Each doubling of preoperative HALP was associated with a lower expected ICU-day rate in the negative binomial model (IRR, 0.718; 95% CI, 0.588–0.878; *p* = 0.001), with a similar estimate in the robust Poisson model (IRR, 0.737; 95% CI, 0.611–0.890; *p* = 0.002). In contrast, preoperative HALP was not significantly associated with total hospital length of stay (negative binomial IRR, 0.951; 95% CI, 0.857–1.056; *p* = 0.348).

In an unadjusted analysis, any intraoperative transfusion was associated with a higher frequency of Clavien–Dindo grade II or higher complications (79.3% vs. 48.0%; OR, 4.153; 95% CI, 1.558–11.069; *p* = 0.003), whereas associations with major complications, anastomotic leakage, and ICU readmission were not statistically significant.

## 4. Discussion

This study evaluated the perioperative trajectory of the hemoglobin–albumin–lymphocyte–platelet (HALP) score and its association with postoperative outcomes following esophagectomy. Three principal findings emerged from this study. First, HALP declined markedly immediately after surgery, reflecting the profound physiological impact of esophagectomy, and showed only partial recovery by POD7. Second, although perioperative HALP dynamics differed according to postoperative outcomes, patients who developed major complications, anastomotic leakage, or unplanned ICU readmission demonstrated a persistently impaired recovery pattern, with significantly lower POD7 HALP values than those with an uncomplicated postoperative course. Finally, longitudinal analyses demonstrated that postoperative HALP, particularly at POD7, showed greater apparent discrimination than the preoperative value alone, remaining independently associated with major complications, anastomotic leakage, and ICU readmission after adjustment for potential confounders. These findings extend evidence on HALP beyond its preoperative role, without establishing prospective prediction of postoperative events. Rather than simply reflecting baseline physiological reserve, postoperative HALP appears to capture the integrated effects of surgical stress, inflammatory response, nutritional status, and early recovery, thereby providing a dynamic assessment of postoperative resilience after esophagectomy.

The marked decline in HALP immediately after esophagectomy is biologically consistent with the integrated physiological response to major surgical trauma rather than reflecting isolated deterioration in nutritional status. Surgical injury triggers a coordinated inflammatory and immunological cascade characterized by the simultaneous activation of pro-inflammatory and anti-inflammatory pathways, with early elevations in circulating cytokines accompanied by transient postoperative immunosuppression. Recent longitudinal immune profiling has further demonstrated that esophagectomy elicits one of the most pronounced postoperative immune perturbations among major surgical procedures, with delayed recovery of immune function being associated with an increased susceptibility to postoperative infectious complications [13].

Concurrently, serum albumin declines rapidly during major oncological surgery. Prospective intraoperative data have shown that abdominal cancer surgery is associated with a substantial reduction in circulating albumin within the first postoperative hours, a phenomenon that reflects the combined effects of the acute inflammatory response, increased vascular permeability, perioperative blood loss, and hemodilution rather than an abrupt deterioration in nutritional reserve [14]. Consistent with this systemic response, major gastrointestinal surgery is also accompanied by a significant decline in circulating T cells, B cells, and natural killer cells during the first postoperative week, highlighting the profound but transient impairment of both innate and adaptive immunity induced by surgical stress [17].

Because the HALP score integrates hemoglobin, albumin, lymphocyte, and platelet parameters into a single immune–nutritional biomarker, perioperative alterations affecting these individual components are expected to collectively influence postoperative HALP values. Recent evidence from a systematic review and meta-analysis including more than 9000 patients with digestive system cancers further supports the concept that HALP reflects the combined nutritional, inflammatory, hematological, and immune status of the host rather than any single biological pathway [11]. Accordingly, the profound decline in HALP observed on postoperative day 1 in our cohort is best interpreted as a dynamic manifestation of the acute postoperative stress response rather than an isolated indicator of malnutrition. This concept is further supported by emerging evidence demonstrating that the prognostic value of HALP may depend not only on its absolute value but also on its temporal evolution during postoperative recovery.

The interpretation of POD7 HALP requires consideration of the potential bidirectional relationship between postoperative physiological recovery and complications. Persistently low HALP may reflect an exaggerated or unresolved systemic response to surgical injury, delayed immune–nutritional recovery, or both. However, postoperative complications may themselves contribute to reductions in HALP through ongoing inflammation, altered albumin concentrations, and changes in circulating blood-cell counts. Thus, lower POD7 HALP may represent not only impaired recovery but also the biological consequences of complications that have already developed. Because the onset of complications could not be reliably established relative to the timing of POD7 blood sampling, the present study cannot distinguish these possibilities or establish that HALP deterioration preceded the clinical events.

An important finding of the present study was that postoperative HALP, particularly on postoperative day 7, demonstrated a stronger association with adverse postoperative outcomes than the preoperative value. This observation suggests that a single preoperative measurement primarily reflects baseline immune–nutritional status, whereas postoperative HALP may additionally capture the evolving physiological response to surgical stress and early postoperative recovery. Nevertheless, the stronger association observed at POD7 should not be interpreted as evidence of superior prospective prediction, because the temporal relationship between HALP measurement and complication onset could not be reliably established. Rather, POD7 HALP may provide an integrated marker of the patient’s immune–nutritional state and clinical course during the first postoperative week. In the comparison with age and ASA alone, the incremental discrimination associated with adding HALP remained statistically significant after Holm correction only for ICU readmission (*p* = 0.0129).

Our findings are supported by recent evidence demonstrating that dynamic changes in immune–nutritional status during treatment may be more informative than pretreatment measurements. In patients with esophageal squamous cell carcinoma receiving neoadjuvant chemotherapy, Hasegawa et al. reported that pre-treatment HALP was not associated with overall survival, whereas a low post-treatment HALP score was significantly associated with worse long-term outcomes. Moreover, the authors concluded that deterioration in nutritional status during treatment, rather than baseline nutritional status alone, was associated with poorer prognosis, emphasizing the importance of serial nutritional assessment throughout the treatment course [18]. These observations parallel our findings despite the different clinical settings, suggesting that the prognostic significance of HALP may depend not only on the patient’s baseline condition but also on how successfully immune and nutritional homeostasis is preserved during major therapeutic stress.

The biological plausibility of this concept is further supported by perioperative immune studies demonstrating that postoperative immune recovery continues throughout the first postoperative week. Persistent impairment of lymphocyte subsets and delayed restoration of adaptive immune function have been associated with prolonged hospitalization and postoperative complications following major gastrointestinal surgery [13,17,19]. Therefore, HALP measured on postoperative day 7 is likely to reflect both the magnitude of the initial surgical insult and the patient’s capacity for early physiological recovery, providing a more comprehensive assessment of postoperative resilience than a preoperative measurement alone. Accordingly, postoperative day 7 appears to represent a clinically meaningful transition point at which persistent impairment of HALP is less likely to reflect the universal physiological response to surgery and more likely to be observed in patients with a complicated postoperative course. This concept is consistent with the growing recognition that the prognostic value of immune–nutritional biomarkers is determined not only by baseline physiological reserve but also by their temporal evolution throughout treatment and postoperative recovery, a principle that has increasingly emerged in recent HALP literature [11,18].

Previous studies have consistently established the prognostic significance of the HALP score across a wide spectrum of solid malignancies, particularly gastrointestinal cancers, where pretreatment HALP has been associated with improved long-term survival outcomes. The concept was first introduced by Chen et al. in gastric cancer, who demonstrated that HALP independently predicted overall survival and improved prognostic discrimination beyond conventional TNM staging through incorporation into a prognostic nomogram [16]. Subsequent studies extended these observations to esophageal cancer. Feng et al. reported that preoperative HALP was an independent predictor of cancer-specific survival in patients undergoing curative resection for esophageal squamous cell carcinoma, while Yamamoto et al. confirmed its independent prognostic value for both overall and recurrence-free survival after curative treatment [8,9]. More recently, two meta-analyses further reinforced the robustness of these findings by demonstrating that low pretreatment HALP was consistently associated with inferior survival across gastrointestinal malignancies and specifically in esophageal and gastroesophageal junction cancers [11,12]. However, despite this growing body of evidence, the available literature has predominantly evaluated HALP as a static pretreatment biomarker.

In contrast to previous studies that primarily investigated pretreatment HALP in relation to long-term oncological outcomes, the present study evaluated serial HALP measurements in the perioperative period and their associations with early postoperative morbidity after esophagectomy. Our findings extend the existing literature by characterizing HALP trajectories across the preoperative period and the first postoperative week, rather than relying on a single baseline measurement. POD7 HALP was associated with overall and major complications, anastomotic leakage, and unplanned ICU readmission, although the retrospective design and incomplete information on complication timing preclude conclusions regarding prospective prediction. Thus, the principal contribution of this study is the characterization of perioperative HALP dynamics and their relationship with the early postoperative clinical course.

The potential clinical relevance of HALP depends not only on its association with postoperative outcomes but also on whether it improves discrimination beyond routinely available clinical variables. In our comparison of age- and ASA-based models, adding POD7 HALP increased the apparent AUC for all four outcomes; however, after Holm correction for multiple comparisons, the improvement remained statistically significant only for unplanned ICU readmission (adjusted *p* = 0.0129). This finding suggests that POD7 HALP may provide additional discriminative information for identifying patients who experience an unplanned return to intensive care, although the magnitude and clinical utility of this improvement require further evaluation. For the other outcomes, the observed AUC increases did not remain statistically significant after correction, and their clinical importance should therefore not be overstated. Moreover, these results do not establish that HALP-guided monitoring or intervention improves patient outcomes. Given the retrospective design, limited sample size, and uncertainty regarding the timing of complications relative to blood sampling, prospective multicenter studies with external validation are needed before HALP can be incorporated into postoperative risk-stratification strategies.

Esophagectomy remains one of the most physiologically demanding gastrointestinal procedures, with postoperative outcomes being strongly influenced by patients’ nutritional reserve and the magnitude of the perioperative inflammatory response. Accordingly, contemporary perioperative guidelines emphasize that nutritional risk assessment should be performed routinely, with early identification of high-risk patients enabling timely nutritional optimization and individualized perioperative management [2,4]. Similarly, recent reviews have highlighted that readily available inflammatory and nutritional biomarkers should be incorporated into perioperative risk assessment because they provide practical information for identifying patients who may benefit from closer surveillance and targeted interventions rather than serving solely as prognostic indicators [3]. In this context, HALP offers several practical advantages for perioperative practice, as it is calculated from routinely available laboratory parameters without additional cost or specialized testing. Rather than replacing established clinical assessment or ERAS pathways, serial perioperative HALP measurements warrant prospective evaluation before they are used to guide intensified surveillance or earlier diagnostic evaluation. This concept is further supported by recent evidence demonstrating that deterioration in perioperative nutritional status is associated with adverse postoperative outcomes following esophagectomy, reinforcing the importance of dynamic rather than isolated preoperative assessment [18]. Likewise, pooled prospective data suggest that persistent postoperative immune dysregulation is closely associated with infectious complications, supporting the rationale for serial monitoring of immune–nutritional status during postoperative recovery [13].

This study has several strengths. Rather than relying on a single pretreatment measurement, we evaluated HALP longitudinally at clinically relevant perioperative time points. Repeated perioperative measurements enabled evaluation of recovery trajectories rather than isolated biomarker values, providing insight into the temporal evolution of postoperative immune–nutritional status. In addition, we analyzed clinically meaningful postoperative outcomes, including major complications, anastomotic leakage, and unplanned ICU readmission, using longitudinal statistical modeling to better characterize the temporal relationship between perioperative HALP changes and adverse events. Nevertheless, several limitations should be acknowledged. The retrospective single-center design may have introduced selection bias and limits the generalizability of our findings. All analyzed operations were performed by one surgical team. The clinical investigators reported that the surgical team changed after 2025 and that subsequent records were less consistently documented; these differences limit comparability with later practice and generalizability to other surgical teams. Although significant associations were identified, external validation in larger multicenter cohorts is warranted. We did not establish an optimal postoperative HALP threshold for clinical decision-making, and our findings should therefore be interpreted as hypothesis-generating.

The exclusion of patients who died or required reoperation before POD7, together with the exclusion of patients lacking complete longitudinal laboratory measurements, may have introduced selection bias and limited the representativeness of the study cohort. In particular, excluding patients with death or reoperation before POD7 may have underrepresented the most severe early postoperative courses. Accordingly, the findings may not be generalizable to patients who experience such early adverse events.

Furthermore, because the temporal relationship between POD7 blood sampling and the onset of postoperative complications could not be consistently established, lower POD7 HALP values may partly reflect the consequences of complications that had already developed. Therefore, the observed associations should be interpreted as relationships with the postoperative clinical course rather than definitive evidence of prospective predictive performance. An event-free POD7 landmark analysis was not possible because the dates of anastomotic leakage and major-complication onset were incompletely documented. These same-sample analyses also cannot establish a clinical benefit from HALP-guided monitoring.

The relatively small number of events for anastomotic leakage and unplanned ICU readmission limited the number of covariates that could be included in the multivariable models. Although parsimonious models were used to mitigate overfitting, residual model instability cannot be excluded. Firth penalization yielded HALP estimates close to those obtained from conventional logistic regression for anastomotic leakage and ICU readmission, but it does not eliminate the uncertainty associated with limited event counts. Internal validation using 1000 bootstrap resamples provides an assessment of model optimism but does not replace external validation.

Tumor categories and stage groups were obtained from postoperative pathological reports and classified according to the eighth edition of the AJCC TNM classification for post-neoadjuvant pathological staging (ypTNM). The recorded stage groups were concordant with the corresponding T, N, and M categories, and these data were summarized descriptively. Given the small numbers of anastomotic-leakage and ICU-readmission events, stage variables were not included in the multivariable models; therefore, residual confounding by disease extent remains possible. Details of neoadjuvant treatment regimens and certain operative factors were also unavailable, leaving the possibility of residual confounding. The detailed diagnostic criteria underlying recorded anastomotic-leakage events could not be independently verified. A single Clavien–Dindo category was available per patient, but the underlying records were insufficient to verify whether it consistently represented the highest complication grade. In addition, the broad grade ≥II outcome includes complications less severe than grade ≥ III events. Accordingly, the adjusted associations should be considered exploratory and require confirmation in larger, independent cohorts.

Finally, long-term oncological outcomes were beyond the scope of the present study. Future prospective studies incorporating serial perioperative HALP assessment, external validation, and clinically applicable cutoff values are needed to determine whether dynamic HALP monitoring can be integrated into routine perioperative risk stratification after esophagectomy.

## 5. Conclusions

Perioperative HALP demonstrates a distinct temporal pattern following esophagectomy, characterized by a marked decline immediately after surgery and partial recovery during the first postoperative week. While early postoperative reductions appear to reflect the expected physiological response to major surgical stress, persistent impairment of HALP by postoperative day 7 was associated with major complications, anastomotic leakage, and unplanned ICU readmission. These findings indicate an association between perioperative HALP patterns and recorded postoperative morbidity; their utility for earlier detection or monitoring decisions has not been established. Prospective multicenter studies are warranted to validate these observations and to establish whether serial HALP monitoring has a clinical role in perioperative management following esophagectomy.

## Figures and Tables

**Figure 1:**
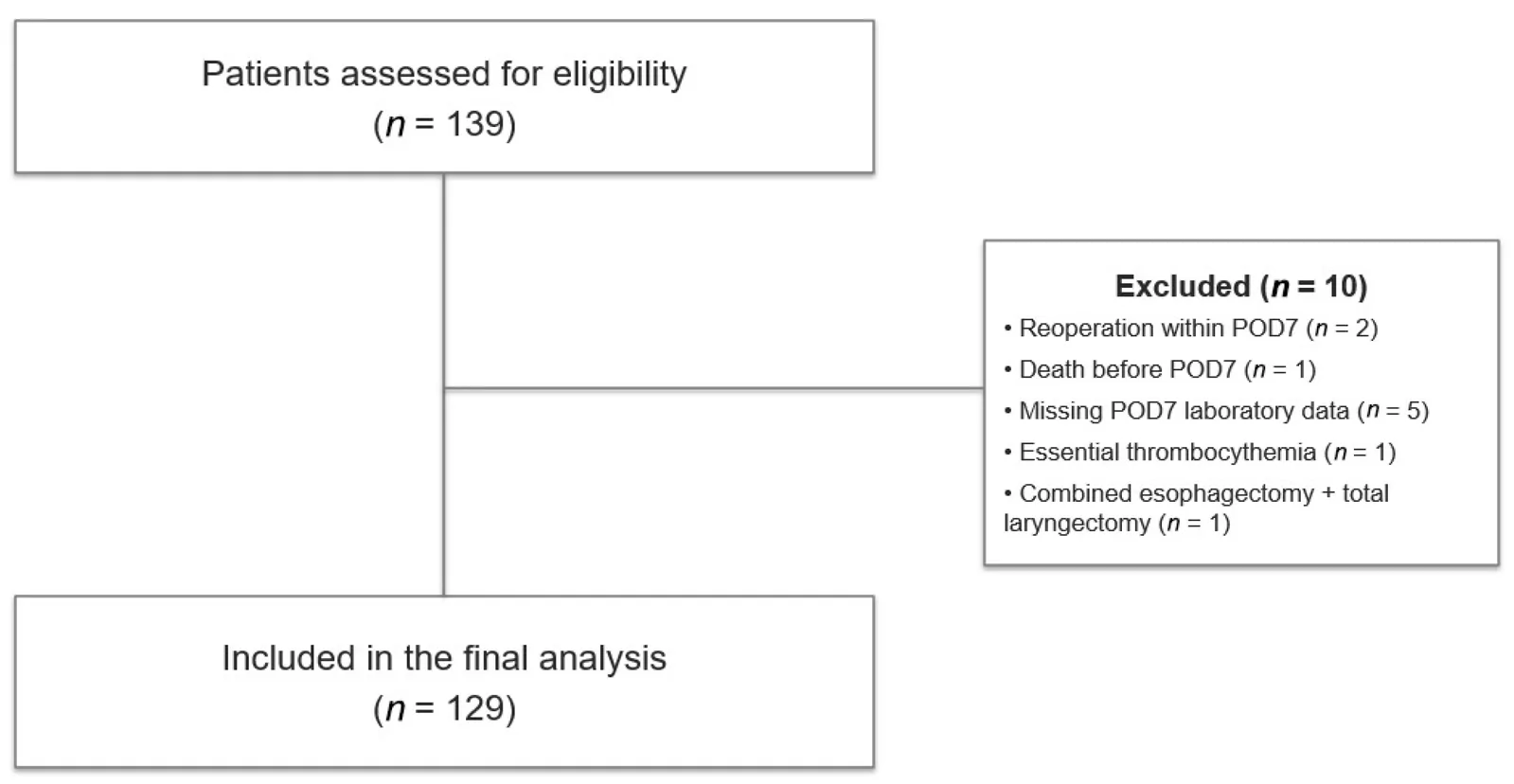
Flow diagram of patient selection showing eligibility assessment, exclusion criteria, and the final study population.

**Figure 2:**
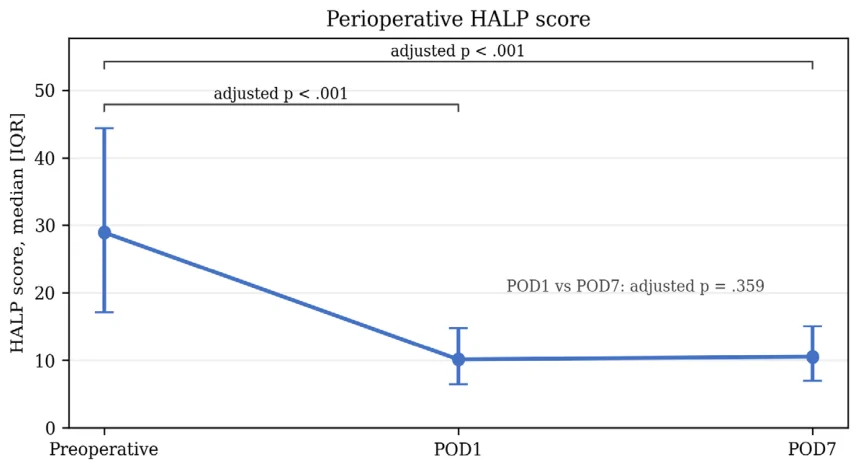
Perioperative changes in HALP score from the preoperative period to POD1 and POD7. Points indicate median values and error bars indicate interquartile ranges. Bonferroni-adjusted pairwise comparisons are preoperative versus POD1, preoperative versus POD7, and POD1 versus POD7.

**Figure 3:**
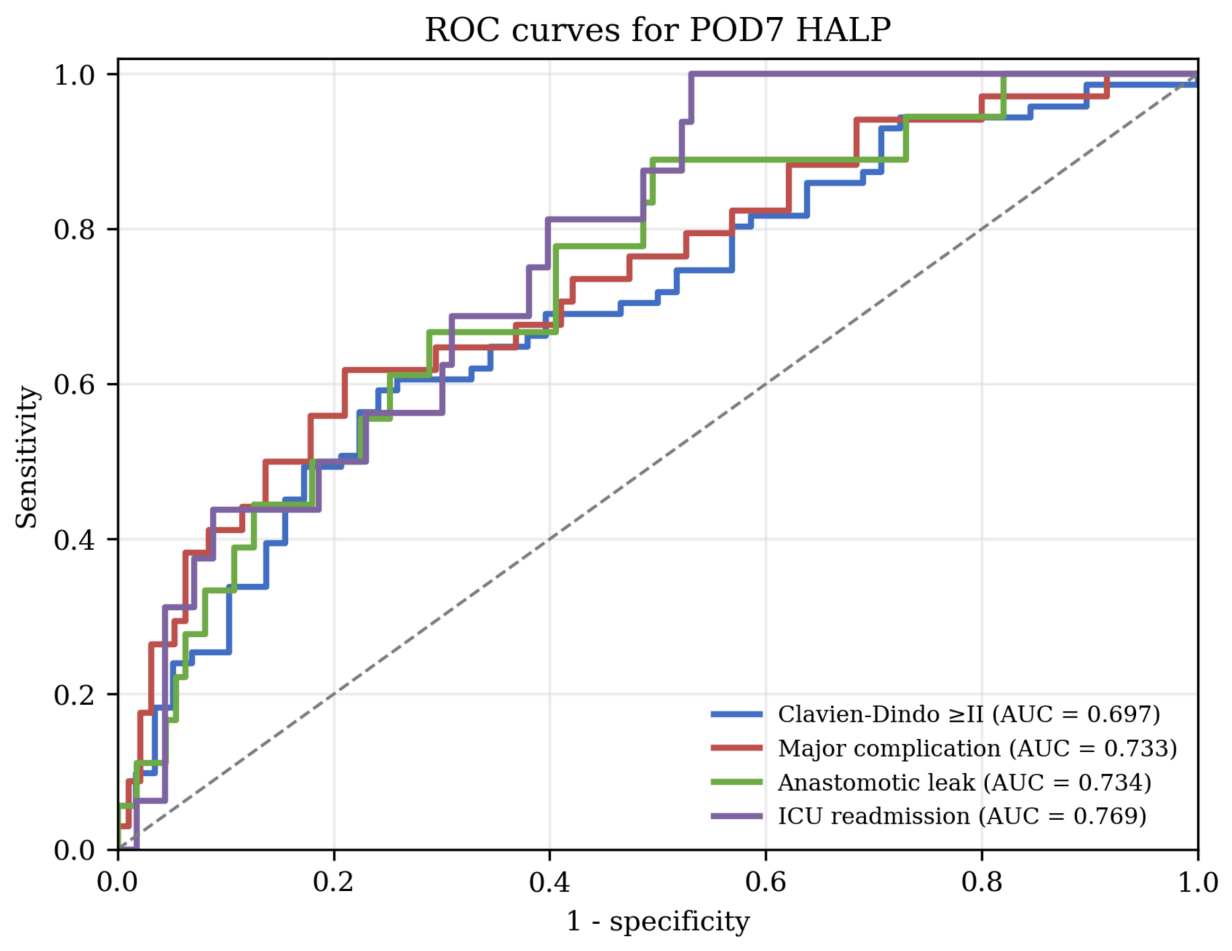
Receiver operating characteristic curves showing the unadjusted discriminatory performance of POD7 HALP alone for Clavien-Dindo grade II or higher complications, major complications, anastomotic leakage, and ICU readmission. The corresponding areas under the curve were 0.697, 0.733, 0.734, and 0.769, respectively. The diagonal line represents chance discrimination, with lower POD7 HALP values indicating greater likelihood of adverse outcomes.

**Figure 4:**
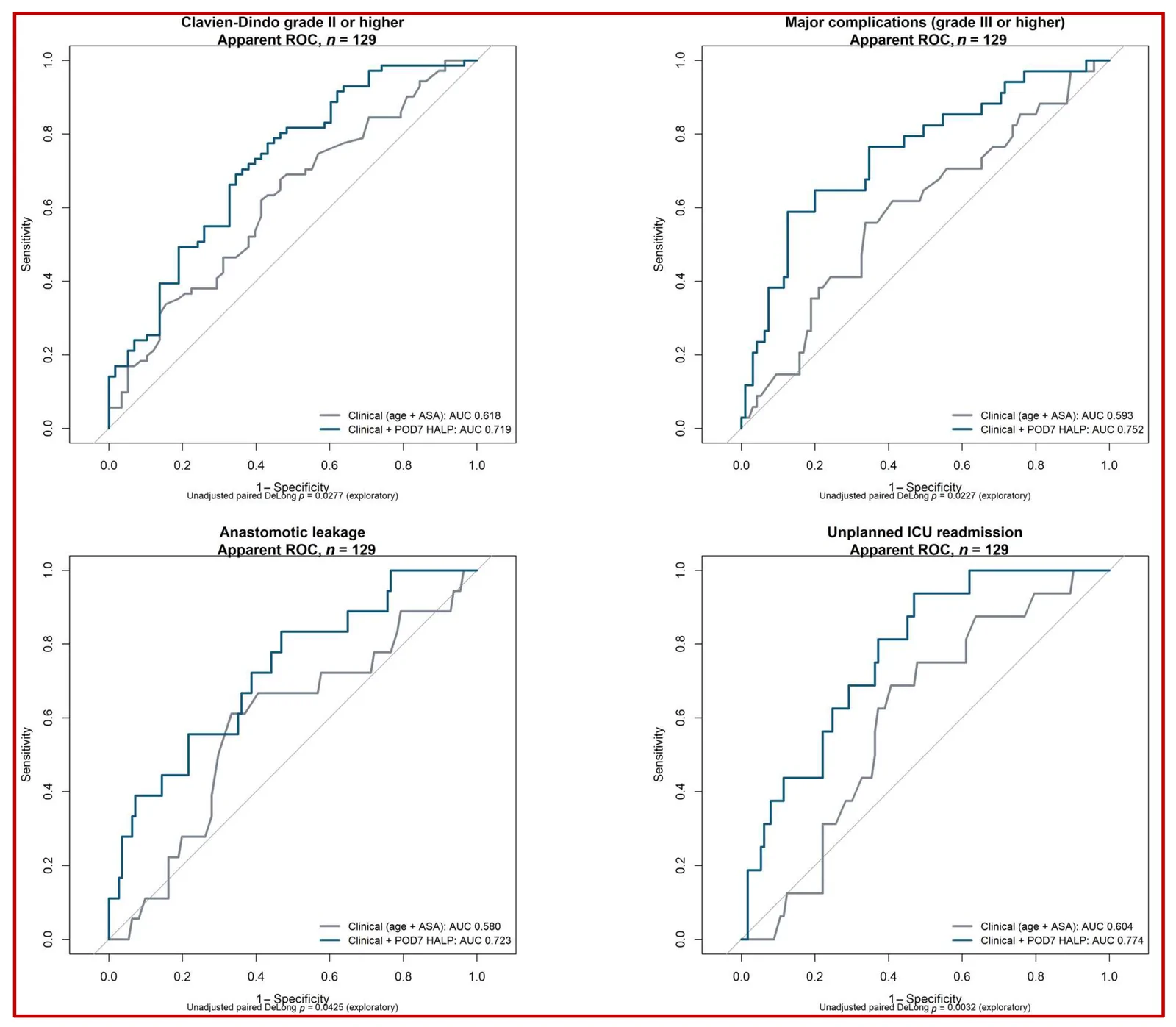
Apparent ROC curves for age- and ASA-based models with and without POD7 HALP (*n* = 129). DeLong *p* values in the panels are unadjusted; Holm correction was applied to the four comparisons (Table 7).

**Table 1:** Demographic, operative, and postoperative characteristics.

Characteristic	Value
Age, years	58.48 ± 12.12
Sex, male/female	63 (48.8)/66 (51.2)
BMI, kg/m^2^	23.72 ± 4.95
ASA I/II/III/IV	4 (3.1)/77 (59.7)/45 (34.9)/3 (2.3)
Recorded yp T0/T1/T2/T3/T4 (*n* = 128)	47 (36.7)/19 (14.8)/13 (10.2)/42 (32.8)/7 (5.5)
Recorded yp N0/N1/N2/N3 (*n* = 128)	82 (64.1)/26 (20.3)/11 (8.6)/9 (7.0)
Recorded yp M0 (*n* = 129)	129 (100.0)
Recorded yp Stage I/II/III (total)/IVA (*n* = 128)	64 (50.0)/19 (14.8)/32 (25.0)/13 (10.2)
Stage III subgroups: IIIA/IIIB/III (NOS)	21 (16.4)/10 (7.8)/1 (0.8)
Surgery: Ivor Lewis/McKeown/transhiatal	49 (38.0)/69 (53.5)/11 (8.5)
Surgery duration, minutes	339.59 ± 89.05
Transfusion: none/ES/FFP/ES + FFP	100 (77.5)/14 (10.9)/5 (3.9)/10 (7.8)
Any intraoperative transfusion	29 (22.5)
ICU length of stay, days	2 [1–3]
Total hospital stay, days	19 [13–30]
Clavien-Dindo grade I/II/III/IV/V	58 (45.0)/37 (28.7)/24 (18.6)/4 (3.1)/6 (4.7)
Clavien-Dindo grade ≥ II	71 (55.0)
Major complication, grade ≥ III	34 (26.4)
Severe complication, grade ≥ IV	10 (7.8)
Anastomotic leak	18 (14.0)
ICU readmission	16 (12.4)
In-hospital death	6 (4.7)

Values are mean ± SD, median [interquartile range], or *n* (%). ES = erythrocyte suspension; FFP = fresh frozen plasma; BMI = body mass index; ASA = American Society of Anesthesiologists physical status; ICU = intensive care unit. Severe complications and in-hospital death were not modeled because only 10 and 6 events were observed. T, N, M, and AJCC eighth-edition post-neoadjuvant pathological (ypTNM) stage-group labels are reported as supplied by the clinical team; the stage labels were not independently re-calculated from T/N/M values. The T1 category comprises T1a (*n* = 5) and T1b (*n* = 14); T4 comprises T4a (*n* = 5) and T4b (*n* = 2). T, N, and stage-group entries were missing for one patient; stage III had no specified subgroup in one patient. For T, N, and stage groups, percentages use 128 nonmissing entries. The IIIA, IIIB, and III (NOS) subgroup counts are included in the stage III total (*n* = 32), not additional patients.

**Table 2:** HALP score and laboratory components across perioperative time points.

Variable	Preoperative	POD1	POD7	χ^2^(2)	*p*	Kendall W
HALP score	28.93 [17.09–44.40]	10.11 [6.48–14.76]	10.53 [6.93–15.02]	145.690	<0.001	0.565
Hemoglobin, g/dL	11.70 [10.60–12.70]	11.10 [9.90–12.10]	10.10 [9.20–11.40]	57.637	<0.001	0.223
Albumin, g/L	41.00 [38.00–44.00]	33.00 [30.00–36.00]	31.00 [28.00–35.00]	171.763	<0.001	0.666
Lymphocyte, 10^9^/L	1.42 [0.90–1.88]	0.54 [0.40–0.83]	0.81 [0.55–1.14]	149.501	<0.001	0.579
Platelet, 10^9^/L	220.0 [180.0–265.0]	203.0 [170.0–257.0]	240.0 [200.0–319.0]	29.020	<0.001	0.112

Values are median [interquartile range]. Overall time effects were evaluated using Friedman tests.

**Table 3:** Adjusted GEE analyses of perioperative log2 HALP trajectories.

Outcome	Interaction χ^2^(2)	Interaction *p*	Preoperative Ratio [95% CI]	*p*	POD1 Ratio [95% CI]	*p*	POD7 Ratio [95% CI]	*p*
Clavien-Dindo ≥ II	3.823	0.148	0.811 [0.641–1.026]	0.081	0.787 [0.626–0.988]	0.039	0.646 [0.513–0.813]	<0.001
Major complication	10.981	0.004	0.940 [0.704–1.256]	0.677	0.699 [0.541–0.905]	0.006	0.549 [0.420–0.720]	<0.001
Anastomotic leak	13.870	0.001	0.964 [0.767–1.211]	0.753	0.934 [0.700–1.246]	0.642	0.555 [0.406–0.760]	<0.001
ICU readmission	8.291	0.016	0.740 [0.551–0.994]	0.045	0.898 [0.643–1.253]	0.526	0.544 [0.414–0.716]	<0.001

Models included categorical time, outcome status, time-by-outcome terms, age, sex, and ASA, with patient-level repeated measurements and exchangeable correlation. Ratios are adjusted geometric-mean HALP ratios for the outcome-present group relative to the outcome-absent group at each time point; values below 1 indicate lower HALP in the outcome-present group. All models included 129 patients with 387 measurements. POD7 (time category 3) was the reference category in the coefficient parameterization. Estimated exchangeable correlations were 0.351, 0.365, 0.383, and 0.368 for Clavien–Dindo ≥ II, major complications, anastomotic leakage, and ICU readmission, respectively.

**Table 4:** Paired comparisons of POD7 HALP AUC with alternative HALP measures.

Outcome	Comparator	POD7 AUC	Comparator AUC	AUC Difference [95% CI]	Raw *p*	Holm-Adjusted *p*
Clavien-Dindo ≥ II	Preoperative HALP	0.697	0.590	0.106 [−0.003–0.216]	0.057	0.344
Clavien-Dindo ≥ II	POD1 HALP	0.697	0.589	0.107 [0.018–0.197]	0.019	0.152
Clavien-Dindo ≥ II	POD7/preoperative ratio	0.697	0.592	0.105 [0.008–0.203]	0.034	0.241
Major complication	Preoperative HALP	0.733	0.537	0.197 [0.066–0.328]	0.003	0.030
Major complication	POD1 HALP	0.733	0.633	0.101 [−0.023–0.225]	0.110	0.350
Major complication	POD7/preoperative ratio	0.733	0.681	0.052 [−0.047–0.151]	0.305	0.611
Anastomotic leak	Preoperative HALP	0.734	0.510	0.225 [0.076–0.373]	0.003	0.030
Anastomotic leak	POD1 HALP	0.734	0.512	0.223 [0.077–0.369]	0.003	0.030
Anastomotic leak	POD7/preoperative ratio	0.734	0.708	0.026 [−0.072–0.124]	0.603	0.611
ICU readmission	Preoperative HALP	0.769	0.644	0.125 [−0.010–0.260]	0.070	0.350
ICU readmission	POD1 HALP	0.769	0.535	0.233 [0.096–0.370]	<0.001	0.010
ICU readmission	POD7/preoperative ratio	0.769	0.643	0.126 [−0.016–0.267]	0.082	0.350

AUCs were compared using paired DeLong tests. Holm adjustment was applied across the 12 displayed comparisons. After Holm adjustment, POD7 HALP showed significantly greater discrimination than preoperative HALP for major complications and anastomotic leakage, greater discrimination than POD1 HALP for anastomotic leakage, and greater discrimination than POD1 HALP for ICU readmission. Other paired AUC differences were not statistically significant.

**Table 5:** Adjusted logistic regression models for POD7 HALP.

Outcome	Adjusted-Model Predictor	Crude OR [95% CI]	Crude *p*	Adjusted OR [95% CI]	Adjusted *p*	Events/N	Model LR *p*	Nagelkerke R^2^
Clavien-Dindo ≥ II	POD7 HALP, per doubling	0.482 [0.315–0.737]	<0.001	0.473 [0.299–0.746]	0.001	71/129	<0.001	0.260
	Preoperative HALP, per doubling			0.927 [0.601–1.428]	0.730			
	Age, per year			0.974 [0.938–1.012]	0.180			
	Female vs. male			0.657 [0.284–1.522]	0.327			
	ASA class, per category			1.736 [0.782–3.852]	0.175			
	BMI, per kg/m^2^			1.019 [0.938–1.106]	0.656			
	Any transfusion vs. none			3.922 [1.319–11.628]	0.014			
Major complication	POD7 HALP, per doubling	0.351 [0.208–0.591]	<0.001	0.351 [0.199–0.619]	<0.001	34/129	<0.001	0.287
	Preoperative HALP, per doubling			1.155 [0.700–1.906]	0.572			
	Age, per year			0.982 [0.944–1.021]	0.351			
	Female vs. male			0.320 [0.123–0.832]	0.019			
	ASA class, per category			0.758 [0.305–1.882]	0.551			
Anastomotic leak	POD7 HALP, per doubling	0.392 [0.217–0.707]	0.002	0.383 [0.210–0.700]	0.002	18/129	0.008	0.160
	Age, per year			1.002 [0.958–1.049]	0.921			
	ASA class, per category			0.650 [0.213–1.977]	0.447			
ICU readmission	POD7 HALP, per doubling	0.374 [0.201–0.695]	0.002	0.374 [0.201–0.696]	0.002	16/129	0.006	0.175
	Age, per year			0.994 [0.948–1.043]	0.815			
	ASA class, per category			1.822 [0.600–5.534]	0.290			

HALP effects are expressed per doubling. The Clavien-Dindo grade II or higher model included preoperative HALP, age, sex, ASA, BMI, and transfusion; the major-complication model included preoperative HALP, age, sex, and ASA; sparse-outcome models included age and ASA. The transfusion estimate is presented as any transfusion versus none. AUC values are apparent same-sample estimates. The preoperative HALP coefficient in models also containing POD7 HALP is distinct from the estimate in a separate preoperative-HALP model. Each adjusted model includes the covariates listed within its outcome-specific block in Table 5; the intercept is not displayed.

**Table 6:** Descriptive comparison of included and excluded patients.

Characteristic	Included (*n* = 129)	Excluded (*n* = 10)
Age, years, mean ± SD	58.48 ± 12.12	59.20 ± 10.61
Male, *n* (%)	63 (48.8)	5 (50.0)
BMI, kg/m^2^, mean ± SD	23.72 ± 4.95	22.91 ± 3.38
ASA II, *n* (%)	77 (59.7)	6 (60.0)
ASA III, *n* (%)	45 (34.9)	4 (40.0)
Ivor Lewis surgery, *n* (%)	49 (38.0)	5 (50.0)
McKeown surgery, *n* (%)	69 (53.5)	5 (50.0)
Surgery duration, min, mean ± SD	339.59 ± 89.05	374.50 ± 67.88
Preoperative HALP, median [IQR]	28.93 [17.09–44.40]	24.53 [21.19–42.96]
POD1 HALP, median [IQR]	10.11 [6.48–14.76] (*n* = 129)	22.38 [11.36–26.21] (*n* = 9)
POD7 HALP, median [IQR]	10.53 [6.93–15.02] (*n* = 129)	12.44 [6.03–19.70] (*n* = 4)
Clavien–Dindo grade ≥ II, *n* (%)	71 (55.0)	7 (70.0)
Major complications (grade ≥ III), *n* (%)	34 (26.4)	4 (40.0)
Anastomotic leakage, *n* (%)	18 (14.0)	4 (40.0)
Unplanned ICU readmission, *n*/available (%)	16/129 (12.4)	0/9 (0.0)
In-hospital death, *n* (%)	6 (4.7)	1 (10.0)

Values are mean ± SD, median [IQR], or *n* (%), as specified. The POD1 and POD7 HALP summaries use available measurements; one excluded patient lacked POD1 HALP and six lacked POD7 HALP. ICU readmission status was missing for one excluded patient. Among the 10 excluded, eight were eligible for the preoperative/POD1 sensitivity analysis; two had a hematological disorder or non-standard surgery. The small and heterogeneous excluded group is described without inferential testing.

**Table 7:** Incremental discrimination from addition of POD7 HALP to age and ASA.

Outcome	Clinical AUC	Clinical + HALP AUC	DeLong *p*	Holm-Adjusted *p*	Corrected + HALP AUC
Clavien–Dindo ≥ II	0.618	0.719	0.0277	0.0680	0.698
Major complications	0.593	0.752	0.0227	0.0680	0.730
Anastomotic leakage	0.580	0.723	0.0425	0.0680	0.696
ICU readmission	0.604	0.774	0.0032	0.0129	0.737

AUC values represent apparent same-cohort discrimination (*n* = 129). Holm *p* values refer to the four paired DeLong tests; corrected AUCs are from 1000-resample bootstrap internal validation. These analyses assess recorded complications, not event-free POD7 prediction. Optimism-corrected Brier scores (clinical model versus clinical + POD7 HALP) were 0.247 versus 0.225 for grade ≥ II complications, 0.201 versus 0.175 for major complications, 0.124 versus 0.114 for anastomotic leakage, and 0.113 versus 0.107 for ICU readmission.

## Data Availability

The data presented in this study are available on reasonable request from the corresponding author. The data are not publicly available due to privacy and ethical restrictions.
